# A review on modern prosthodontic practice using laser

**DOI:** 10.6026/973206300200946

**Published:** 2024-08-31

**Authors:** Queen Alice Arul, Dipanjan Debnath, Padmapriya Mahalingam

**Affiliations:** 1 Department of Dentistry, AIIMS (All India Institute Of Medical Sciences), Kalyani, West Bengal, India; 2 Department of Conservative Dentistry & Endodontics, Government Dental College, Chennai, Tamil Nadu, India

**Keywords:** Lasers, fixed prosthetics, removable prosthetics, implantology, aesthetic dentistry, maxillofacial prosthodontics

## Abstract

Lasers are a very appealing technology in many areas of dentistry because of their minimally invasive nature, quick tissue interaction & response, and ability to heal. Numerous investigations on the possible uses of lasers in dentistry have been carried out since Maiman developed the ruby laser in 1960. Numerous applications such as quick prototyping and computer-aided design, and the study of occlusion in complete denture using three-dimensional laser scanning has been used to develop dentures. Its uses are numerous from fixed prosthodontics to dentinal hypersensitivity treatment to surface treatment of base metal alloys. These days, it even encompasses maxillofacial prosthodontics and dental implantology. Hence, it is of interest to review the use of lasers in clinical prosthodontics.

## Background:

A specialized sector has emerged in the field of dentistry as a result of the rapid development of lasers and their wavelengths with a variety of applications during the past few decades. There have been certain challenges with conventional dental procedures that have been overcome by use of laser technology. Therefore, it is of interest to report about the applications of laser in different perspectives of prosthodontics. "Light Amplification by Stimulated Emission of Radiation" is what the abbreviation "LASER" stands for, and the term itself highlights the key component of laser action. [1] In 1964, American scientist Charles Hard Townes presented the fundamental ideas of laser technology for the first time. The first person to show a laser in an experiment was Theodore H. Maiman of California, who did it in 1960 by projecting light through a ruby crystal. [1] These days, patients want the best outcomes with the minimum efforts. When used properly, lasers in prosthodontics can shorten the time between appointments, improve up healing, and produce better outcomes. Every laser device has the following parts- a) A medium, which m ay consist of a gas, liquid, or solid. The laser is called after this medium, which controls the wavelength of light emitted by the device. b) A laser tube or optical cavity with two mirrors at either end-one entirely reflecting and the other partially transmissive-that makes up the cavity. c) An external power source of a particular sort. The atoms in the laser medium are excited, or "pumped," to greater energy levels by the external power source. [2, 3] There is a wide range of wavelengths available to dentists for laser application in the oral cavity. A thorough comprehension of how these various laser wavelengths interact with one another and the target tissues are necessary for the best possible treatment outcomes. The various domains where laser can be used are described following.

## Fixed prosthodontics:

## Crown lengthening:

Lasers provide the best control over the procedure, accurately shaping the gingival margin and finely tracing the incision lines. Erbium laser treatment results in minimum tissue displacement for osseous crown lengthening. [5]

## Teeth preparation:

The best method for preparing dental hard tissues is to use an Er:YAG laser. Anaesthetic is typically not necessary because the Er:YAG laser numbs the tooth in the majority of cases. There is even no chance of enamel micro-fractures when using a laser as compared to using a high-speed handpiece. [6]

## Trough formation:

Prior to taking an impression, a trough is formed around the tooth structure. This eliminates the requirement for hemostatic drugs, electrocautery, and the use of retraction cords. The laser minimizes post-operative pain and chair time by providing a quick, predictable, no or minimal bleeding during impression. [7]

## Veneer removal:

The water molecules in the adhesive absorb the laser energy as it passes through the ceramic glass. At the silane-resin interface, de-bonding takes place. Depending on the thickness of the ceramic restoration, the process takes two seconds to two minutes. [8]

## Preparing e-models:

Since e-models are created from scanned impressions, a 3D laser scanner is a useful tool because it is simple to use and eliminates the need for casting preparation. It is made up of a visible light laser beam and a triangulation-based camera-like device. [9]

## Removable prosthodontics:

## Exostoses or tori removal:

If the mucosal covering gets ulcerated or if the exostoses are larger or irregular in shape, it may result in difficulties for the prosthesis fabrication. To remove the bony protuberances, erbium: yttrium- aluminium-garnet (Er: YAG) is the primary therapeutic option. The primary benefits of using an Er: YAG laser are less overheating, decontamination, bio-stimulation, and the lack of smear layer formation, which impedes the healing process. [10]

## Fibroma:

Sharp denture flanges or excessive pressure on the posterior palatal seal region might result in fibrous tissue formation and long-term tissue damage. Any soft tissue laser can be used to treat this, allowing the tissue to re-epithelialize. [11]

## Vestibuloplasty:

When a narrow vestibule length and mandibular or maxillary crest atrophy contribute to poor prosthetic stability, vestibuloplasty is the treatment of choice. CO2 technology offers a safe and easy way. There is no need for the grafts or sutures. A soft acrylic relining must be applied to removable dentures immediately and for a limited time. Following surgery, patients should wear the denture for three to four weeks.[12]

## Frenectomy:

To improve denture retention, high labial frenulum (lateral or median) should be excised since it may lead to instability of the oral prosthetic structure. [13] A CO2 laser can accomplish the goal.

## Epulis fissuratum:

In individuals with CDs, epulis is an excessive growth of mucosa due to persistent tissue irritation. The most popular methods for removing the lesion are surgical or electrical scalpels, and soft tissue lasers. [14]

## Denture stomatitis:

Between 60% and 65% of people who wear dentures are susceptible to this persistent candidal infection. In addition to aiding in the ablation of the epithelium surface contaminated by candida, a laser beam also helps by preventing inflammation of the nearby normal mucosa. Antibiotics or non-steroidal anti-inflammatory medicines (NSAIDs) won't need to be prescribed after surgery because the laser has virucidal and bactericidal properties of its own. Because of their neuron sealing effect, lasers provide sufficient pain alleviation. [15]

## To assess the accuracy of the impression and complete denture occlusion:

The recently introduced laser scanner is a 3D digitizer that records data at a resolution of 130 mm at 100 mm by tracking the specimen's coordinates (x, y, and z). Complex 3D texture-mapped models are captured by the 3D laser and sent into a 3D program called Scan Surf, where they are constructed and triangulated to create a 3D meshwork image of the target.[16]

## Making dentures:

Selective laser sintering is used to fabricate dentures (SLS). In laser fusion, metal or polymer powders are melted at a high temperature using a high intensity CO2 laser beam. Consequently, it creates the strongest and longest-lasting design form from a digital model that is saved in a standard triangulation language (STL) format. [17] The component of the removable partial dentures that is laser welded: The fabrication of the prosthesis is done with a Nd: YAG laser. Comparing laser welding to soldering, 20%-50% greater tensile strength values can be reached. [18]

## Implantology:

## Preparing the implant site:

Removing any overlying structure promotes quicker healing, improved integration, less discomfort for the patient, and more bone-to-implant contact.[19]

## Second stage uncovering:

Soft tissue that is overlaying is removed using a CO2 laser and nearly all other types of lasers. Because there is less blood contamination and tissue shrinkage during second stage surgery, an immediate impression can be taken.

## Peri-implantitis:

Granulated tissue at the inflammatory site can be removed, and the implant surface can be disinfected using a diode, CO2, or Er: YAG laser.[20]

## Repairing an ailing implant:

After applying toluidine blue O for a minute, contaminated surfaces are exposed to LLLT, such as diode soft lasers (690 nm), for 60 seconds. The number of bacteria is lowered by 92%.

## Aesthetic dentistry:

## Smile design:

At the present time, pulsed Nd: YAG, erbium, diode, and argon lasers are utilized for this purpose. Soft tissues are outlined with a pencil before utilizing a laser to ensure that the gingival contour, symmetry, axial tilt, and zenith are all correct. To assess the marginal gingiva, a periodontal probe is positioned apically to the alveolar crest. The biological width is identified and marked with a laser to serve as a finishing point and point of reference, enabling the doctor to concentrate on aesthetics. [21]

## Bleaching:

Ar and diode lasers are frequently used for in-office bleaching procedures. As of right now, the only laser bleaching system having photothermal, photochemical, and photocatalytic activity is the KTP laser and H2 O2 gel combination (Smart Bleach gel [SBI]). The goal of laser teeth whitening is to achieve maximum bleaching effect without experiencing any negative side effects.

## Maxillofacial prosthodontics:

## 3D Printing:

This technique, which creates models from computer-aided designs (CADs) that are constructed layer by layer, has become indispensable for both soft and bone tissue reconstruction. In the context of therapeutic settings, several 3D printing methods, including stereolithography, fused deposition modelling, multi jet modelling, SLS, binder jetting, and binder jetting, have been investigated [23, 24].

## Conclusion:

A laser technique is quicker, less painful, and causes little to no discomfort, which reduces tissue damage in the surrounding area. It is an outstanding and efficient technique that may be applied to both hard and soft tissues. To optimize the number of treatments in dental practice, clinical practitioners and dentists should be aware about the various laser systems.

## Funding:

The authors did not receive funds or grants, or other support from any organization for the submitted work.

## Disclosure statement:

The authors report there are no competing interests to declare.

## Authors Contribution:

All the authors contributed equally in drafting the manuscript.

## Ethical Statement:

Not applicable as it does not involve any human or animal subjects.

## Figures and Tables

**Figure 1:**
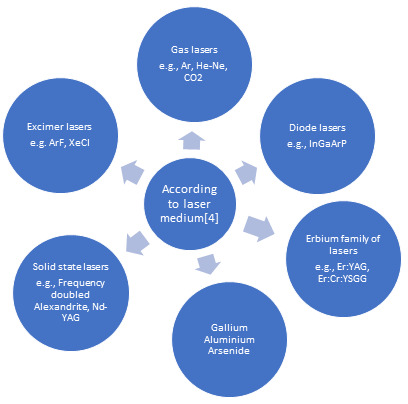
Classification of lasers [4]
